# Altered estradiol-dependent cellular Ca^2+^ homeostasis and endoplasmic reticulum stress response in Premenstrual Dysphoric Disorder

**DOI:** 10.1038/s41380-021-01144-8

**Published:** 2021-05-25

**Authors:** Howard J. Li, Allison Goff, Sarah A. Rudzinskas, Yonwoo Jung, Neelima Dubey, Jessica Hoffman, Dion Hipolito, Maria Mazzu, David R. Rubinow, Peter J. Schmidt, David Goldman

**Affiliations:** 1 Dept. of Obstetrics, Gynecology & Reproductive Sciences, Yale School of Medicine, New Haven, CT USA; 2 Section on Behavioral Endocrinology, National Institute of Mental Health, NIH, Bethesda, MD USA; 3 Laboratory of Neurogenetics, National Institute of Alcohol Abuse and Alcoholism, NIH, Bethesda, MD USA; 4 Dept. of Psychiatry, University of North Carolina, Chapel Hill, NC USA

**Keywords:** Cell biology, Neuroscience, Genetics, Psychiatric disorders, Molecular biology

## Abstract

Premenstrual Dysphoric Disorder (PMDD) is characterized by debilitating mood symptoms in the luteal phase of the menstrual cycle. Prior studies of affected women have implicated a differential response to ovarian steroids. However, the molecular basis of these patients’ differential response to hormone remains poorly understood. We performed transcriptomic analyses of lymphoblastoid cell lines (LCLs) derived from women with PMDD and asymptomatic controls cultured under untreated (steroid-free), estradiol-treated (E2), and progesterone-treated (P4) conditions. Weighted gene correlation network analysis (WGCNA) of transcriptomes identified four gene modules with significant diagnosis x hormone interactions, including one enriched for neuronal functions. Next, in a gene-level analysis comparing transcriptional response to hormone across diagnoses, a generalized linear model identified 1522 genes differentially responsive to E2 (E2-DRGs). Among the top 10 E2-DRGs was a physically interacting network (*NUCB1*, *DST, GCC2*, *GOLGB1*) involved in endoplasmic reticulum (ER)-Golgi function. qRT-PCR validation reproduced a diagnosis x E2 interaction (F(1,24)=7.01, *p* = 0.014) for *NUCB1*, a regulator of cellular Ca^2+^ and ER stress. Finally, we used a thapsigargin (Tg) challenge assay to test whether E2 induces differences in Ca^2+^ homeostasis and ER stress response in PMDD. PMDD LCLs had a 1.36-fold decrease in Tg-induced *XBP1* splicing response compared to controls, and a 1.62-fold decreased response (*p* = 0.005), with a diagnosis x treatment interaction (F(3,33)=3.51, *p* = 0.026) in the E2-exposed condition. Altered hormone-dependent in cellular Ca^2+^ dynamics and ER stress may contribute to the pathophysiology of PMDD.

## Introduction

Premenstrual Dysphoric Disorder (PMDD) affects 3–8% of reproductive-aged women and involves debilitating mood symptoms confined to the luteal phase of the menstrual cycle [1, 2]. Symptoms occur after ovulation and remit shortly after menses, suggesting a role for luteal hormones. While peripheral levels of estradiol, progesterone, and other reproductive steroids are normal in PMDD [3, 4], clinical hormone manipulations suggest PMDD is a disease induced by physiologic fluctuations in reproductive hormones. Women with PMDD experience remission of mood symptoms after pharmacologic suppression of the hypothalamic-pituitary-gonadal (HPG) axis with GnRH agonists and recurrence when estradiol (E2) or progesterone (P4) are blindly added back—a response not seen in healthy controls [5]. Neuroimaging studies have demonstrated both persistent and hormone-induced differences in neurocircuitry and cognition in PMDD, including emotional processing, corticolimbic control, and working memory [6, 7].

Recent evidence suggests these physiologic characteristics of PMDD may be underpinned by genetic and cellular factors. Using lymphoblastoid cell lines (LCLs), the ESC/E(Z) complex, a set of 13 genes involved in epigenetic silencing via H3K27 methylation, was found to be differentially expressed in PMDD via transcriptomic analysis in the baseline, hormone-free condition [8]. While ESC/E(Z) expression differences could serve as a potential substrate for an abnormal response to hormone, the molecular and functional correlates of a differential hormone response (including the transcriptional consequences of altered ESC/E(Z) complex expression) have not been fully characterized.

In this study, we first explore the transcriptomic basis of differential steroid response in PMDD using a module-level analysis to identify differentially regulated gene modules and a gene-level analysis to identify differentially regulated genes. Informed by these results, we designed an in-vitro assay to test whether there was a hormone-dependent functional difference in Ca^2+^ homeostasis and ER stress response between PMDD and control LCLs.

## Methods

### Participants

Women with PMDD and asymptomatic controls were recruited as previously described [8]. Briefly, participants were 18-48 years old, had regular menses (21–35 days), and were not medically ill, taking medications, or pregnant. DSM-5 criteria were prospectively confirmed through daily symptom ratings over three menstrual cycles [9]. Women underwent clinical interview and self-report questionnaire to confirm diagnostic criteria. Additionally, each woman had symptom ratings (irritability, depression, anxiety, mood lability) ≥30% increased (relative to the range of the symptom scale) in the week before menses, compared to the week after. Women with significant symptoms outside the luteal phase were excluded.

In controls, absence of symptoms was confirmed using daily ratings over 2 months. Women contributing samples for RNAseq underwent a trial of leuprolide and hormone add-back [8]. Women in the PMDD RNAseq group experienced symptom remission with leuprolide and recurrence with E2 or P4 add-back using defined criteria [5]. The control group remained euthymic throughout the entire protocol. An independent cohort of women with PMDD and controls who otherwise met study inclusion criteria but did not undergo leuprolide/add-back trials contributed samples for experimental validation. Participant demographics summarized in Supplemental Table S1. This study was approved by the NIH IRB. All participants provided written informed consent according to NIH intramural guidelines.

### Cell culturing, hormone treatments, and RNA sequencing

LCLs were generated via Epstein-Barr Virus transformation of mononuclear cells from peripheral blood [10]. LCLs at 2 × 10^5^ cells/mL in phenol-free RPMI (Gibco, cat. 11835-055) with 15% KOSR (Gibco, cat. 10828-028), 2% glutamine (200 mM), and 1% gentamicin (5 mg/ml) were cultured for 3–5 days at 37 °C with 5% CO_2_ before treatment with estradiol (100 nM, Sigma-Aldrich, cat. E2758-5G) or progesterone (100 nM, Sigma-Aldrich, cat. P8783-25G) for 24 h, or no treatment. Full details are given in Dubey et al. [8]. Cells were harvested for RNAseq, described in Supplemental Methods. Workflow of data generation and analysis is summarized in Supplemental Fig. S1.

### Module-level analysis: WGCNA, GSEA, and Enrichr

Weighted Gene Correlation Network Analysis (WGCNA) was conducted using R v.1.66 [11]. Modules were generated from CPM values of 46 samples analyzed by RNAseq. To identify modules differentially responsive to hormone in PMDD, two-way ANOVA was performed on module eigengenes, and modules showing a diagnosis x hormone interaction (nominal *P* < 0.05) were considered differentially responsive. Functional analysis of modules was performed using Gene Set Enrichment Analysis (GSEA) [12]. In a secondary analysis, core subsets underlying top GSEA enrichment signals were defined using Leading Edge Analysis (LEA), and a consensus set was used for pathway analysis under Enrichr tools [13]. Full details are given in Supplemental Methods.

### Gene-level analysis: EdgeR, GeneMANIA, and qRT-PCR

Generalized linear modeling of RNAseq data within EdgeR identified genes differentially regulated by either E2 (E2-DRGs) or P4 (P4-DRGs) [14]. Each set of paired RNAseq samples (untreated and E2-treated, and untreated and P4-treated) was used to fit a separate model of hormone response. Paired samples with Trimmed Mean of M-Values (TMM)-normalized counts and tagwise dispersion estimation were fitted to a quasi-likelihood negative binomial generalized log-linear model. Factors of subject (cell line) and treatment (untreated vs. hormone-treated) were nested under diagnosis (control vs. PMDD). Genewise F-tests were performed with each model to identify genes showing significant diagnosis x hormone interactions (nominal *P* < 0.05), yielding a list of E2-DRGs and P4-DRGs.

Network visualization was performed in GeneMANIA, a web-based tool that accepts an unranked gene list and generates a weighted interaction network based on published databases [15]. Select genes were validated in a biologically independent cohort of cases and controls via TaqMan qRT-PCR (Applied Biosystems), described in Supplemental Methods.

### Thapsigargin challenge assays

A thapsigargin (Tg) challenge assay compared the response of PMDD and control LCLs to Tg, a sarco/endoplasmic reticulum Ca^2+^-ATPase (SERCA) channel inhibitor, under untreated and E2-treated conditions. LCLs were seeded at 2 × 10^5^ cells/mL in phenol red-free RPMI, divided into 5 ml cultures in 6-well plates, and cultured for 3–5 days. For E2 pre-treatments, E2 in DMSO was added for a final concentration of 100 nM and cultured for 20 h. An equivalent volume of DMSO served as vehicle control. For Tg treatments, Tg (DMSO) and CaCl_2_ (aq) were added for a final concentration of 0.2 uM and 0.8 uM, respectively, and cultured for an additional 4 h. RNA extraction and cDNA synthesis were performed as described above. Spliced-to-unspliced *XBP1* ratios (*s/u XBP1*), a marker of ER stress response activation, were quantified via qRT-PCR, described in Supplemental Methods.

### Statistical analysis

Statistical analyses were performed in GraphPad Prism v8 (La Jolla, CA) and R v3.4.1 (Vienna, Austria). Student’s *t* test and Fisher’s Exact tests were conducted for demographic variables. qRT-PCR analyses were performed with repeated measures ANOVA with Sidak’s multiple comparisons.

## Results

### WGCNA identifies modules associated with abnormal hormone response

**Fig. 1:**
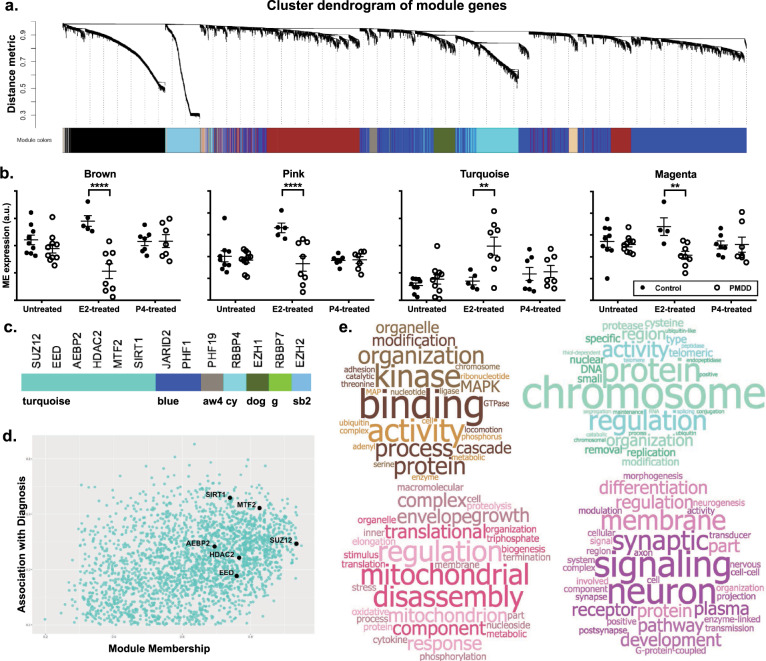
Functional analysis of gene modules from hormone-exposed PMDD LCLs. **a** Cluster dendrogram of gene modules. In WGCNA analysis of 46 input transcriptomes, 21,746 unique gene features (“genes”) were clustered into 65 co-expression modules using gene–gene correlation patterns across all input transcriptomes. **b** Module eigengenes showing significant diagnosis x hormone interactions. Eigengenes (defined as the first principal component of a given module) of four modules (Brown, Pink, Turquoise, and Magenta) showed differential regulation by hormone (ANOVA interaction *P* < 0.05). Closed circles denote controls. Open circles denote PMDD. Note: An extreme outlier in the E2-treated control group was excluded in the eigengene plot of the Magenta module for scaling purposes. Hormone x diagnosis interaction was significant both before and after exclusion of this outlier. *P*-values are not adjusted for multiple comparisons. **c** Module membership of ESC/E(Z) complex genes. Nearly half (6/13) of ESC/E(Z) complex genes were assigned to the Turquoise module. Remaining ESC/E(Z) genes were clustered into the Blue, AntiqueWhite4 (aw4), DarkOliveGreen (dog), Cyan (cy), Green (g), and SkyBlue2 (sb2) modules. **d** ESC/E(Z) complex genes in the Turquoise module. Genes plotted according to module membership and strength of association with diagnosis. ESC/E(Z) complex genes (*SUZ12, EED, AEBP2, HDAC2, MTF2, SIRT2*) are more likely to have high module membership within the Turquoise module. **e** Word cloud representations of GSEA results. Top 20 GSEA results were used to generate word clouds for each differentially regulated module. Word size is proportional to frequency of word among GO terms identified by GSEA results. Modules were enriched for terms relating to MAP Kinase and GTPase signaling (Brown, top left), chromatin remodeling and proteasome function (Turquoise, top right), mitochondrial function (Pink, bottom left), and neuronal and synaptic function (Magenta, bottom right). Full, quantitative GSEA results are tabulated in Supplemental Table S3. Word clouds generated via worditout.com.

Of 26,362 genes represented in 46 input libraries, 22,242 (84%) were sufficiently expressed for WGCNA, and 21,746 (98%) clustered into 65 modules (Fig. 1a). Four modules (arbitrarily designated Turquoise, Pink, Brown, and Magenta) had eigengenes with significant diagnosis x hormone interactions (Fig. 1b, Supplemental Table S2).

All 13 ESC/E(Z) complex genes clustered into modules with significant effects of diagnosis, hormone, or their interaction (Fig. 1c). Nearly half (*SIRT1, MTF2, AEBP2, HDAC2, SUZ12, EED*) were high-connectivity members of the Turquoise module. *SUZ12* had the fourth highest connectivity by module membership among 2492 module genes (Fig. 1d).

In functional GSEA analysis of significant modules, Turquoise was enriched for terms relating to chromatin remodeling, proteasome function, and transcriptional processing. Pink was enriched for mitochondrial function. Brown contained many terms for MAP Kinase and GTPase signaling. Magenta was enriched for neuronal and synaptic function (Fig. 1e). Complete GSEA results in Supplemental Table S3.

### Top E2-DRGs comprise a physically interacting network involved in ER–Golgi function and Ca^2+^ homeostasis

Linear modeling of RNAseq data in EdgeR identified 1522 genes differentially responsive to E2 (E2-DRGs) and 480 differentially responsive to P4 (P4-DRGs). Full EdgeR results in Supplemental Tables S4, S5.

**Fig. 2:**
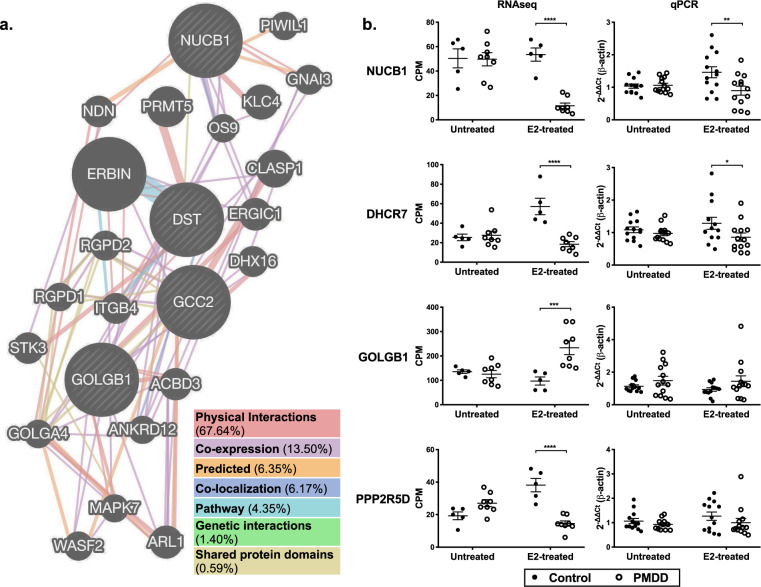
Top E2-DRGs implicate ER–Golgi function and Ca^2+^ homeostasis in PMDD. **a** GeneMANIA visualization of select E2-DRGs. Key genes relating to ER–Golgi function and intracellular calcium homeostasis (*NUCB1, DST, GCC2, GOLGB1*) were selected from among the top 10 E2-DRGs and included as inputs for GeneMANIA network visualization (genemania.org, accessed January 2019). The resulting output revealed a structural network of Golgi–ER-associated genes, with physical interactions comprising 67.6% of the weighted network. **b** qRT-PCR validation of select E2-DRGs. *NUCB1, DHCR7, PPP2R5D*, and *GOLGB1* were selected for qRT-PCR validation in an independent replication cohort of 13 cases and 13 controls. NUCB1 qRT-PCR results revealed a significant main effect of diagnosis (F(1,24)=4.61, *p* = 0.042) and interaction of diagnosis and hormone (F(1,24)=7.01, *p* = 0.014). In post-hoc testing, E2-treated PMDD samples had significantly reduced *NUCB1* expression compared to control samples (*p* = 0.003). qRT-PCR results for *DHCR7* did not reveal significant effects of diagnosis, hormone, or their interaction, but in multiple comparisons testing, DHCR7 expression was significantly reduced in E2-treated PMDD samples compared to E2-treated control samples (*p* = 0.040). No significant differences for *PPP2R5D* or *GOLGB1* were found in qRT-PCR validation.

We focused on the E2-treated condition because: (1) E2-treatment appeared to drive the diagnosis x hormone interaction seen in modules of interest (Fig. 1b); (2) There were no P4-DRGs with FDR < 0.25 (Supplemental Table S5); (3) Hierarchical clustering of transcriptomes showed stronger clustering among E2-treated samples compared to P4-treated samples, and greater segregation between cases and controls within the E2-treated cluster (Supplemental Fig. S2a); and (4) Modules of interest tended to be enriched for E2-DRGs rather than P4-DRGs (Supplemental Fig. S2b, Supplemental Table S2).

Table 1 lists the top 10 E2-DRGs. Several of these genes are directly related to cellular Ca^2+^ handling and the ER–Golgi compartment (*NUCB1*, *DST*, *GCC2*, and *GOLGB1*), and were included as inputs for GeneMANIA network visualization. The resulting output revealed a network of Golgi–ER-associated genes, with physical interactions contributing 67.6% to the weighted network (Fig. 2a).

**Table 1:** Top 10 genes differentially regulated by E2 in PMDD.

Gene	Name	Description^a^	logCPM	F	P-Val
***NUCB1***	Nucleobindin 1	Member of a small calcium-binding EF-hand protein family. Thought to have a key role in Golgi calcium homeostasis and Ca(^2+^)-regulated signal transduction.	5.31	28.99	4.28E-05
***DST***	Dystonin	Member of the plakin protein family of adhesion junction plaque proteins. Some isoforms expressed in neural and muscle tissue, anchoring neural intermediate filaments to actin.	8.52	24.13	1.17E-04
*MDH2*	Malate Dehydrogenase 2	Mitochondrial enzyme catalyzing the reversible oxidation of malate to oxaloacetate, utilizing the NAD/NADH cofactor system.	7.49	23.07	1.48E-04
*PPP2R5D*	Protein Phosphatase 2 Regulatory Subunit B’Delta	Belongs to the phosphatase 2 A regulatory subunit B family. One of the four major Ser/Thr phosphatases. Implicated in the negative control of cell growth and division. Cause of autosomal dominant intellectual disability.	4.61	22.27	1.78E-04
*DHCR7*	7-Dehydrocholesterol Reductase	Catalyzes the conversion of 7-dehydrocholesterol to cholesterol. Localizes to the ER membrane and nuclear outer membrane. Associated with Smith–Lemli–Opitz syndrome.	4.91	21.95	1.91E-04
***GCC2***	GRIP and Coiled-Coil Domain Containing 2	Peripheral membrane protein localized to the trans-Golgi network. Contains a GRIP domain thought to be used in Golgi targeting.	7.05	21.56	2.09E-04
***GOLGB1***	Golgin B1	May participate in intercisternal cross-bridges of the Golgi complex.	7.28	20.57	2.65E-04
*GPI*	Glucose-6-Phosphate Isomerase	Interconverts glucose-6-phosphate and fructose-6-phosphate. Extracellularly, promotes survival of skeletal motor neurons and sensory neurons, and is a lymphokine inducing immunoglobulin secretion.	8.22	20.29	2.84E-04
*PTP4A3*	Protein-Tyrosine Phosphatase 4a3	Member of the protein-tyrosine phosphatase family. Enhances cell proliferation, motility, and promotes cancer metastasis. May be involved in the progression of cardiac hypertrophy by inhibiting intracellular calcium mobilization.	4.99	20.26	2.86E-04
*SLC35A4*	Solute Carrier 35A4	Probable UDP-sugar transporter protein. Paralog of SLC35A2, which transports UDP-galactose into Golgi vesicles, where it serves as a glycosyl donor for the generation of glycans.	5.2	19.87	3.15E-04

Top 10 most significant EdgeR genes differentially regulated by E2 in PMDD. Genes related to intracellular calcium homeostasis, the Golgi–ER compartment, and the ER stress response are bolded (*NUCB1, DST, GCC2, GOLGB1*). Nominal *P* values are reported. All top 10 genes had a FDR < 0.25. Complete statistics are listed in Supplemental Table S4.

^a^Gene descriptions are abstracted from Entrez, GeneCards, UniProtKB entries listed on genecards.org.

Select genes were chosen for qRT-PCR validation in a biologically independent cohort of 13 cases and 13 controls. Repeated measures ANOVA on *NUCB1* qRT-PCR results revealed a main effect of diagnosis (F(1,24)=4.61, *p* = 0.042) and diagnosis x hormone interaction (F(1,24)=7.01, *p* = 0.014) (Fig. 2b).

### E2 induces differences in Ca^2+^ homeostasis and ER stress response in PMDD LCLs

**Fig. 3:**
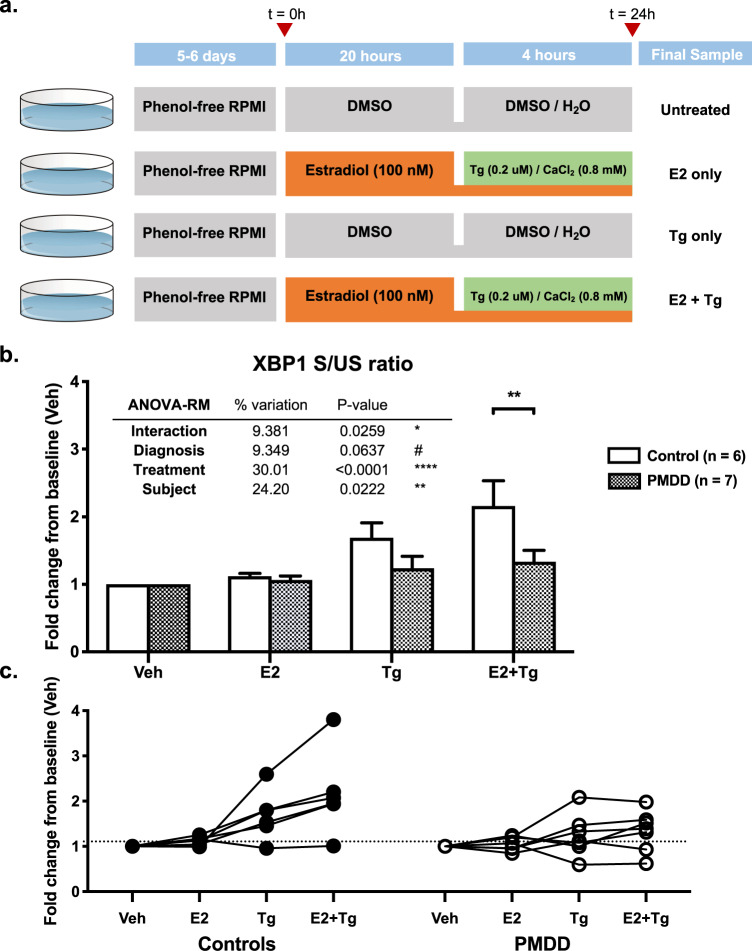
Thapsigargin (Tg) challenge differentiates PMDD and Control LCLs. **a** Experimental design of Tg challenge assays. PMDD and control LCLs were cultured in phenol red-free RPMI for 5–6 days and treated in four arms: (1) no treatment (vehicle), (2) E2 (100 nM) for 24 h, (3) Tg (0.2 uM) and CaCl_2_ (0.8 mM) for 4 h, and (4) E2 (100 nM) for 24 h with Tg (0.2 uM) and CaCl_2_ (0.8 mM) for the last 4 h. Cells were harvested and assayed for the spliced-to-unspliced ratio of *XBP1* by qRT-PCR. **b** XBP1 S/US ratios in Tg challenge assays (by diagnosis group). Control LCLs showed an increase in XBP1 S/US ratio after Tg treatment. Pre-treatment with E2 potentiated this response. Both of these effects were blunted or absent in PMDD LCLs. A significant diagnosis x treatment interaction (ANOVA-RM F(3,33) = 3.508, *p* = 0.026) in *XBP1* splicing response to Tg was observed. Post-hoc analysis revealed a significant difference between control and PMDD LCLs only when cells were treated with the combination of E2 + Tg (*p* = 0.005). E2 had a significant modulatory effect on Tg response in control cells (*p* = 0.007) but not in PMDD cells (*p* = 0.693) (see Supplemental Table S6). **c** S/US XBP1 ratios in Tg challenge assays (by individual cell line). Examining S/US XBP1 ratios of individual cell lines across treatment conditions shows qualitatively distinct trajectories between control and PMDD groups, most notably, the loss of E2-mediated potentiation of *XBP1* splicing in PMDD LCLs. However, significant within-group heterogeneity among cell lines is also appreciated.

Thapsigargin (Tg), a potent SERCA inhibitor, rapidly increases cytosolic Ca^2+^ levels and triggers the ER stress response [16]. We tested the effects of Tg and E2, alone and in combination, and found a significant main effect of treatment (F(3,33)=11.2, *p* < 0.001) and a significant diagnosis x treatment interaction (F(3,33)=3.51, *p* = 0.026). The main effect of diagnosis was itself not significant (*p* = 0.064) (Fig. 3a).

In post-hoc analysis, control LCLs had a 1.7-fold increase in *XBP1* splicing with Tg alone (*p* = 0.009) and a 2.2-fold increase with Tg preceded by E2 (*p* < 0.0001). PMDD LCLs showed a blunted, non-significant increase in *XBP1* splicing in both conditions. Furthermore, while E2 exposure modulates Tg-induced *XBP1* splicing in Control LCLs (*p* = 0.007), addition of E2 did not alter response to Tg in PMDD LCLs (*p* = 0.693). Comparing PMDD and control LCLs within-treatment, *XBP1* splicing in PMDD was 1.36-fold decreased compared to controls in the Tg-only condition (*p* = 0.25). This difference was accentuated (1.62-fold decrease, *p* = 0.005) when Tg was preceded by E2. E2 alone did not affect *XBP1* splicing regardless of diagnosis (Fig. 3b, c). Full ANOVA-RM results in Supplemental Table S6.

### Secondary analysis of a neuronally relevant module implicates Ca^2+^ dynamics and the ESC/E(Z) complex

We revisited our module-level analysis to see if Ca^2+^-related pathways were implicated by Magenta module genes. To explore potential pathways represented by Magenta module hub genes, Leading Edge Analysis (LEA) of top 20 enriched Magenta GO terms with FDR < 0.25 was used to identify a core subset of 88 genes (Supplemental Table S7). Enrichr analysis found that these genes were enriched for GO terms relating to Ca^2+^ dynamics, including Ca^2+^-dependent synaptic vesicle handling and Ca^2+^ ion transport. Surprisingly, these genes were also enriched for targets of ESC/E(Z) complex genes *SUZ12* and *EZH2*, estrogen receptor *ESR1*, and RE1-Silencing Transcription Factor (*REST*), which suppresses expression of neural genes in non-neuronal cell lines [17], and genes associated with the H3K27me3 histone marker. The top enriched KEGG pathways were neuroactive ligand–receptor interaction, the cAMP, Rap1, and Ras pathways, and glutamatergic synapse (Supplemental Fig. S3). Full Enrichr results are given in Supplemental Table S8.

## Discussion

PMDD is a disorder of atypical response to ovarian steroids [5]. While we recently reported differences in ESC/E(Z) expression in hormone-free conditions, the molecular and functional correlates of differential hormone response in PMDD remain poorly defined. Comparing LCLs from PMDD cases and controls, we identified four gene modules differentially responsive to hormone, including one enriched for neuronal functions. Analysis of E2-DRGs suggested a role for altered Ca^2+^ handing and the ER–Golgi system. A functional assay showed both the ER stress response and its normal modulation by E2 were blunted in PMDD. Thus, starting from a global transcriptomic analysis, we report the first indications of a hormone-dependent functional difference in cells of women with PMDD.

Each of four modules implicated via WGCNA represents a plausible pathway in PMDD. Two modules (Blue and Pink) were enriched for MAPK and GTPase signaling and mitochondrial function, respectively. These functions have been implicated in mood disorders including as mediators of sex differences [18–22] and estrogen response [23–25]. The Turquoise module is associated with ESC/E(Z), an epigenetic modifier gene complex recently implicated by our group in PMDD. Isolation of a neuronally relevant module (Magenta) is notable because LCLs are non-neuronal cells. While other LCL-based studies of psychiatric disease identified individual genes of potential neuronal relevance [26–29], with others leveraging WGCNA to implicate non-specific cellular or immunologic modules [30–32], this is the first study using LCLs to implicate a neuronally relevant module in psychiatric disease. In a preliminary analysis, Magenta hub genes were enriched for targets of *REST*, a repressor of neuronal genes in non-neuronal cells [17], suggesting that despite mechanisms limiting neuronal gene expression such as *REST*-mediated transcriptional repression, potentially meaningful differences in aggregate expression could still be detected, perhaps due to careful phenotyping and appropriate clinical stratification.

Our gene-wise analysis implicated the ER–Golgi system and cellular Ca^2+^ dynamics. *NUCB1*, an independently validated E2-DRG, encodes a Golgi protein regulating endomembrane Ca^2+^ uptake and ER stress response [33, 34]. It is also implicated in spatiotemporal Ca^2+^ regulation during neurotransmission [35], with an emerging role in mood and behavior. *NUCB1* is modulated by sex hormones and affects behavior in animal models [36]. Homolog *NUCB2* is a sex-specific correlate of depression, anxiety, and suicide in humans [37–40]. Other top E2-DRGs include *GOLGB1* (encodes a cross-bridging protein of Golgi cisternae), *GCC2* (encodes a molecular tether between transport vesicles and the Golgi), and *DST* (encodes a neuronal isoform critical in ER function) [41–43]. *DST* mutations cause hereditary sensory neuropathy, possibly via Ca^2+^ dyshomeostasis, pathologic ER stress response induction, and subsequent sensory neuron apoptosis [44]. Together, the differential expression of these genes suggests Ca^2+^ homeostasis and its effects on the Golgi–ER system and ER stress could be disrupted in PMDD.

The ER stress response, also known as the unfolded protein response, is a highly conserved response to Ca^2+^ perturbation and protein misfolding: induction of protein-folding chaperones, ribosome halting, upregulated proteolysis, and increased cytoplasmic-endomembrane Ca^2+^ transport [45, 46]. In the LCL model, E2 (rather than P4) was the primary driver of differential hormone response, and was thus used in our functional assays. While P4 is clinically important [47, 48], the primacy of E2 in our RNAseq experiments likely reflects the absence of nuclear P4 receptors in lymphoid cells [8, 49]. On the other hand, nuclear estrogen receptors *ESR1* and *ESR2* are abundantly expressed and mediate estrogen-dependent changes in gene expression [50]. These are distinct from membrane E2 and P4 receptors, which are both expressed in lymphoid cells but are thought to mediate the immunomodulatory effects of E2 and P4 via rapid intracellular signal transduction events rather than transcriptional regulation [51, 52]. Furthermore, while both membrane steroid receptors and nuclear steroid receptors (in their membrane-localized state) likely orchestrate E2- and P4-mediated synaptic remodeling via non-genomic signal transduction events, particularly via G-protein coupled receptors, these effects are less likely to be detected by an RNAseq-based methodology [53–56].

Although control LCLs initiate a Tg-induced ER stress response that is potentiated by E2, both of these effects are diminished or absent in PMDD LCLs. This result parallels similar findings in LCLs from patients with bipolar disorder (BD). So et al. found attenuated *XBP1* induction in BD LCLs compared to controls [57]. Similar findings were later independently reported by Hayashi et al. and Pfaffenseller et al. [58, 59]. Interestingly, BD shares significant phenotypic overlap with PMDD, and PMDD patients are frequently misdiagnosed with BD [60].

While gene-level analysis identified select E2-DRGs related to Ca^2+^ and ER stress, the connection between module-level findings and our observed impairments in ER stress response was less clear. Due to the potential relevance of Ca^2+^ dynamics to neuronal biology, we wondered if a secondary analysis of the Magenta module would implicate Ca^2+^-relevant pathways. As GSEA results suggested broad functional terms (“Synapse”, “Neuron”, “Axon”) without specifically implicating subcellular pathways, we focused on a union set of 88 leading edge (LEA) genes compiled from top GSEA enrichment signals. LEA subsets prioritize high-rank (i.e., high-connectivity) genes, selecting for the most functionally relevant hub genes. In addition to identifying enriched GO terms relating to Ca^2+^ handling, Enrichr analysis of this subset also implicated targets of ESC/E(Z) genes *SUZ12* and *EZH2* and estrogen receptor *ESR1*, suggesting a potential relationship between the ESC/E(Z) complex, estrogen response, and transcriptional control of Magenta module genes. This relationship is reinforced by a concomitant enrichment of histone marker H3K27me3-associated genes; H3K27 is a specific target of ESC/E(Z) [61], and the only known methyltransferase capable of methylation at H3K27me3 is *EZH2*, a core ESC/E(Z) effector [62]. As this implies a regulatory relationship between Magenta and Turquoise modules, both differentially responsive modules in PMDD, we hypothesize that in PMDD, a set of genes involved in neurotransmission, synaptic function, and Ca^2+^ dynamics (represented by the Magenta module) may be abnormally regulated by H3K27 epigenetic remodeling (represented by the Turquoise module and the ESC/E(Z) complex). These putative relationships require further study.

There is some genetic evidence for ER stress genes in neuropsychiatric disease. Among canonical ER stress response mediators *XBP1*, *GRP94* (*HSP90B1*), *GRP78* (*HSPA5*), *CHOP* (*DDIT3*), *PERK* (*EIF2AK3*), *ATF6*, and *IRE1* (*ERN1*), Genome-wide association (GWA) studies identified a *PERK* variant (rs7571971) associated with progressive supranuclear palsy [63], and an *ATF6* variant (rs10918270) associated with Parkinson’s Disease [64]. The plausible implication of ER stress pathways in PMDD by RNAseq suggests that transcriptomics may uncover genetic etiologies not revealed by GWAS, which has only partially explained the liability to affective illnesses.

E2 has been studied as a modulator of ER stress. In breast cancer, E2 has a protective, activating effect on ER stress response. Under mitogenic E2 signaling, cells activate an “anticipatory” ER stress response alongside upregulated protein synthesis and mitosis [65, 66]. In non-dividing cells such as neurons, E2-mediated priming of ER stress pathways might protect against non-mitotic sources of ER stress and Ca^2+^ perturbation—namely, excitatory synaptic input. This is consistent with prior work demonstrating protective effects of estrogen in glutamate excitotoxicity via Ca^2+^ dynamics stabilization [67]. Blunting of this anticipatory homeostatic pathway may increase neuronal excitability and partially explain the observation that women with PMDD demonstrate a relative resistance to the expected inhibitory effects of GABA-A receptor modulators such as allopregnanolone, thought to be a key pathophysiologic mechanism in PMDD [68]. Cellular Ca^2+^ dynamics are also critical in microglial activation, a neuroimmune process perhaps better approximated by LCLs. Dysregulated microglial activation and other neuroinflammatory processes have been implicated in depressive disorders [69, 70]. Finally, a critical end-organelle of ER stress signaling are mitochondria, which play important roles in Ca^2+^ sequestration, ROS metabolism, inflammatory signaling, and apoptosis [71, 72]. Mitochondria were not only implicated in our own WGCNA analysis, but have emerged as mediators of estrogen’s neuroactive effects in psychiatric and neurodegenerative disease, particularly through their role in regulating intracellular Ca^2+^ [24, 71, 73, 74].

Our findings also have therapeutic implications. The ER stress pathway is altered by many mood stabilizing drugs [75], and there is ample clinical precedent for targeting cellular Ca^2+^ in psychiatric disease. Although now supplanted by antidepressants and mood stabilizers, Ca^2+^ channel blockers such as verapamil were once commonly and efficaciously used in the treatment of anxiety, depression, bipolar disorder, and premenstrual symptoms [76, 77]. If confirmed in future studies, hormone-dependent alterations in the ER stress response and Ca^2+^ homeostasis represent a novel yet conceptually plausible pathway in the pathophysiology of PMDD.

### Limitations and future work

We found hormone-dependent differences in Tg-induced ER stress response, but in a modest sample of 13 patient-derived LCLs. Cell lines used in Tg experiments were not completely independent from those used in initial RNAseq experiments. Generalizability of results would be supported by validation in larger, independent cohorts.

Our findings of altered ER stress require mechanistic follow-up. By using *XBP1* splicing as the functional output, we did not investigate Ca^2+^ homeostasis and ER stress as independent processes. Quantifying Ca^2+^ dynamics through calcium imaging or electrophysiology, and measuring other ER stress markers (e.g., *GRP94*, *GRP78*, *CHOP*) or ER stress intermediaries (PERK, ATF6, IRE1) would enable a better mechanistic understanding of altered Ca^2+^ dynamics and ER stress in PMDD. The role of *NUCB1* in modulating ER stress and Ca^2+^—a promising lead given its validation as an E2-DRG—is another area for future experimentation.

The role of ESC/E(Z) in mediating this cellular phenotype should also be investigated. A secondary analysis of key Magenta module hub genes suggested a role for ESC/E(Z) in regulating both neuronal function and cellular Ca^2+^ dynamics. The selection of Leading Edge genes for secondary analysis, while an established method of isolating statistically and functionally important genes within a module, can introduce selection bias. We cannot conclude that this module is the transcriptional basis of altered Ca^2+^ homeostasis. The purported role of ESC/E(Z) on Ca^2+^ dynamics and neuronal biology may be better addressed by multi-omic methods (integration of ChIP-seq and RNAseq datasets) and the use of a more neuronally relevant model (e.g., neuronal cell lines from patient-derived stem cells or postmortem brain tissue), especially as LCLs are in many respects not an authentic model of neuropsychiatric function [78–80]. For PMDD, a particular gap is the absence of nuclear progesterone receptors in LCLs. Furthermore, attribution of our findings to genetic variants or epigenetic markers would extend the understanding of PMDD as a genetically influenced disease.

Finally, despite significant differences in between-group analysis, appreciable within-group variation existed in *NUCB1* expression and ER stress activation in both PMDD and control LCLs. If PMDD is a heterogeneous diagnosis, blunted E2-dependent ER stress activation may be common among both cases and controls, but associated with just one of multiple molecular etiologies resulting in psychiatric vulnerability to luteal phase hormones. Future work to correlate alterations in Ca^2+^ dynamics and ER stress, with clinical features (including response to therapies targeting these pathways) may allow for stratification of this complex diagnosis for research and clinical management.

## Supplementary information

Supplemental Tables

Supplemental legends

Supplemental Methods

Figure S1

Figure S2

Figure S3
