# Supplementary material for: Altered estradiol-dependent cellular Ca2+ homeostasis and endoplasmic reticulum stress response in Premenstrual Dysphoric Disorder
Source: Mol Psychiatry. 2021 May 25;26(11):6963–74. doi: 10.1038/s41380-021-01144-8 (PMC8613306; doi:10.1038/s41380-021-01144-8)
Supplement: Supplementary file 2 — Supplemental legends [file 41380_2021_1144_MOESM2_ESM.docx]

**Supplemental Legends**

**Supplemental Figures**

**Supplemental Figure S1. RNAseq analysis workflow**.

Women contributing samples for LCL generation and RNA sequencing were phenotyped using daily symptom ratings, self-report questionnaire, clinical interview, and trial of leuprolide with E2 and P4 add-back. Patient-derived LCLs were cultured under untreated, E2-treated, and P4-treated conditions and then harvested for RNA sequencing. RNAseq data were analysed using two approaches: (1) In a module-level analysis, Weighted Gene Correlation Network Analysis (WGCNA) identified differentially regulated modules. The functions of these modules were explored for functional enrichment using GSEA. One module of interest (Magenta) was further analyzed using Leading Edge Analysis (LEA) to identify core genes underlying enrichment signals identified in GSEA. LEA genes were analyzed for functional enrichment in Enrichr to discover potential physiologic pathways for downstream experiments. (2) In a gene-level analysis, EdgeR fitted a linear model to RNAseq data to identify individual genes differentially regulated by E2 (E2-DRGs) or P4 (P4-DRGs). Select DRGs were explored with network visualization in GeneMANIA and validated via qRT-PCR. Results from these analyses were then used to inform the design of a physiologic in-vitro assay.

**Supplemental Figure S2. Response to E2, not P4, underlies differential hormone response in PMDD LCLs.**

a. Hierarchical clustering of transcriptomes show the following: 1. E2-treated samples cluster more strongly together than P4-treated samples; and 2. Within the E2-treated cluster, there is greater segregation of samples by diagnosis.

b. Plot of modules by significance of main effect of diagnosis (x-axis), and diagnosis x hormone interaction (y-axis), - log ANOVA P-value. The size of each point is proportional to the enrichment ratio of E2-DRGs (left) or P4-DRGs (right), defined as the observed/expected proportion of DRGs in each module. E2-DRG and P4-DRG enrichment scores for all modules are listed in **Supplemental Table S2**.

**Supplemental Figure S3. Enrichr analysis of Magenta module LEA genes implicates Ca^2+^-relevant pathways in PMDD**

High connectivity magenta module genes isolated by Leading Edge Analysis were inputted into Enrichr for functional enrichment analysis against 6 datasets: **a.** ENCODE and ChEA Consensus TFs from ChIP-X (2016) (red bars) **b.** NIH Roadmap Epigenomics Histone Modification ChIP-seq data set (2015) (brown bars), **c.** KEGG cell signaling pathways (2016) (orange bars), and GO terms for **d.** Molecular Function (blue bars), **e.** Biological Process (purple bars), and **f.** Cellular Component (green bars) (2018). Bars are proportional to Combined Score Ranking, a composite score proportional to the log P-value from a Fisher Exact Test for gene enrichment, and the Z-score deviation from expected rank. Calcium-relevant GO pathways are highlighted.

**Supplemental Tables**

**Supplemental Table S1. Demographics of women in each study component.**

Demographic variables of women with PMDD and healthy controls contributing cell lines are reported for each study component: RNAseq analysis, qRT-PCR validation, and Tg challenge assays. For all study components, no significant differences were found in race/ethnicity, age, BMI, obstetrical history (parity), past psychiatric history (history of major depressive episodes, and history of psychotropic medication use), with the exception of age in the qRT-PCR validation cohort; women in the PMDD group were significantly older than women in the control group by an average of 9.3 years (p = 0.0003). For each study component, women in the PMDD group more frequently had a history of prior major depressive episodes, consistent with the selection criteria for the Control group. This difference was not statistically significant.

**Supplemental Table S2. Diagnosis x hormone treatment interaction on module eigengene expression.** Eigengene values were extracted from all 46 input samples across all 65 modules. Two-way ANOVA of eigengene values tested the effects of Diagnosis, Hormone Treatment, and their interaction. Four modules (Brown, Pink, Turquoise, and Magenta) showed a significant Diagnosis x Hormone interaction (P < 0.05). P-values are unadjusted for multiple comparisons. Module eigengenes are also displayed with corresponding enrichment scores for E2-DRGs and P4-DRGs within each associated module, defined as the ratio of observed vs. expected frequency of DRGs within a given module. Modules associated with a significant diagnosis x hormone interaction are enriched for E2-DRGs more so than P4-DRGs.

**Supplemental Table S3. Top 20 GSEA results for 4 modules with eigengenes showing diagnosis x hormone interaction.**

Module genes were pre-ranked by the WGCNA intramodular connectivity metric to prioritize high-connectivity hub genes, and pre-ranked lists were analyzed against the C5 Gene Ontology gene sets with 1000 permutations. Gene sets enriched at FDR-corrected q-value < 0.25 were considered significant. Word frequencies from names of enriched gene sets were used to generate word clouds shown in **Figure 1e**.

**Supplemental Table S4-S5. EdgeR output for transcripts differentially responsive to E2 or P4 in PMDD LCLs**

In EdgeR, paired RNAseq samples were fitted to a quasi-likelihood negative binomial generalized log-linear model in which the factors of subject (cell line) and treatment (untreated vs. hormone-treated) were both nested under diagnosis (control vs. PMDD). Genewise F-tests were conducted to identify genes showing significant diagnosis x hormone interactions (nominal P < 0.05). This analysis was performed for both E2-treated and P4-treated samples, yielding a list of genes differentially responsive to E2 (E2-DRGs) and to P4 (P4-DRGs).

**Supplemental Table S6. Repeated measures ANOVA analysis of Tg challenge experiments showing blunted XBP1 splicing in PMDD.**

PMDD LCLs exhibit a blunted XBP1 splicing response to Tg compared to control LCLs, and this effect is accentuated when LCLs were pre-treated with estradiol prior to Tg exposure. Analysis of XBP1 splice-to-unspliced ratios show a significant main effect of treatment (F(3,33) = 11.22, P < 0.001), and a significant diagnosis x treatment interaction (F(3,33) = 3.508, P = 0.0259). The effect of diagnosis was nearly significant (F(1,11) = 4.25, P = 0.0637). In post-hoc analysis, control LCLs had a 1.7-fold increase in XBP1 splicing from baseline with Tg alone (p = 0.0085), and a 2.2-fold increase with Tg preceded by E2 (p < 0.0001). In contrast, PMDD LCLs showed a blunted, non-significant increase in XBP1 splicing in both conditions. E2 exposure significantly modulated the *XBP1* splicing response to Tg in Control LCLs (p = 0.007), but did not alter response to Tg in PMDD LCLs (p = 0.693). Comparing PMDD and control LCLs within treatment conditions, the splicing response of PMDD LCLs was 1.36-fold decreased compared to control LCLs in the Tg-only condition (p = 0.25), and this difference was accentuated and significant when Tg was preceded by E2, with a 1.62-fold decreased splicing response (p = 0.005).

**Supplemental Table S7. Leading edge analysis of top 20 Magenta module GSEA GO terms.**

Top 20 GO terms from GSEA analysis of the magenta module were selected for leading edge analysis (LEA). Leading edge analysis of each enriched gene set yielded a core set of high-ranked (high-connectivity) genes that contributed most to the enrichment signal. The union of these 20 core sets (one for each enriched GO term) comprised 88 LEA genes. In this table, these 88 LEA genes are listed, with contributions to each gene set signal listed as “1” or “0” (“1” denoting that the gene contributes to a given gene set signal). Total signal contributions of each gene are also reported, with genes ranked in order of greatest number of signal contributions. Genes symbols and their corresponding gene names are both listed and highlighted in yellow if they were directly noted to be involved in calcium binding, calcium transport, or calcium signaling based on their gene summaries as listed in genecards.org. Gene sets are numbered 1-20, with full names of the gene set listed at the bottom of the table.

**Supplemental Table S8. Enrichr analysis of Magenta LEA genes.**

Magenta LEA genes were inputted into Enrichr for overlap analysis against four databases: 1. ENCODE and ChEA Consensus TFs from ChIP-X, 2. NIH Roadmap Epigenomics Histone Modification ChIP-seq data set, 3. KEGG’s cell signaling pathways (2016), and GO terms for Biological Processes, Molecular Function, and Cellular Component (2018). Magenta LEA genes were enriched for targets of EZH2, SUZ12, and ESR1, as well several pathways related to calcium transport, signaling, and homeostasis. Analysis was performed in December 2018.
