# Supplementary material for: Altered estradiol-dependent cellular Ca2+ homeostasis and endoplasmic reticulum stress response in Premenstrual Dysphoric Disorder
Source: Mol Psychiatry. 2021 May 25;26(11):6963–74. doi: 10.1038/s41380-021-01144-8 (PMC8613306; doi:10.1038/s41380-021-01144-8)
Supplement: Supplementary file 3 — Supplemental Methods [file 41380_2021_1144_MOESM3_ESM.docx]

**Supplemental Methods**

***RNA sequencing***

Total RNA from cell pellets was isolated using TRIzol extraction (Life Technologies, cat. no. 15596018), treated with DNase (Qiagen, cat. no. 79254), and further purified using the RNeasy MinElute Cleanup kit (Qiagen, cat. no. 74204). RNA quality was analyzed on the Agilent 2100 Bioanalyzer, and total RNA was used to prepare cDNA libraries using the Ambion RNAseq library construction kit (Invitrogen, cat. 4454073). Libraries were sequenced on the Illumina Genome Analyzer IIx system. Raw RNAseq reads in SCARF format from Illumina pipeline CASAVA_v1.8.1 were exported as FASTQ reads and aligned to the UCSC hg19 reference genome using TopHat2 using a read segment length of 25, mismatch and gap thresholds of 2, insertion and deletion thresholds of 3, and minimum splice anchor length of 8.^1^ The featureCounts tool in the Subread package was used to generate genewise counts from mapped bam files.^2^ Counts were converted to CPM and RPKM values using an in-house script. Subsequent transcriptomic analysis of RNAseq data is schematically summarized in **Supplemental Figure S1.**

***Module-level analysis: WGCNA, GSEA, and Enrichr***

Weighted Gene Correlation Network Analysis (WGCNA) was conducted using the WGCNA R package v.1.66.^3^ WGCNA uses co-expression patterns between genes across a set of RNAseq samples to discover, in an unsupervised manner, clusters of co-regulated genes (“modules”). Modules were generated from raw CPM values of 46 samples (**Supplemental Table S1**) using an unsigned correlation measure, soft thresholding power of 4, minimum module size of 30, and a merge cut height of 0.25. Discovered modules are assigned colors (e.g. Red, Green, DarkOrange, etc.) as arbitrary, non-ordinal identifiers. To identify differentially responsive modules, values of module eigengenes were extracted from each module across all samples. An eigengene is defined as the first principal component of genes within a given module and used as a robust measure of overall expression activity of that module. Two-way ANOVA analysis was performed on module eigengenes, and modules with eigengenes showing a diagnosis x hormone interaction (nominal p < 0.05) were considered differentially responsive.

Functional analysis of module genes was conducted using Gene Set Enrichment Analysis (GSEA)^4^ and Enrichr tools.^5,6^ In GSEA, pre-ranked module genes were analyzed against the C5 Gene Ontology gene sets downloaded from MSigDB 6.2 Collections^7–9^ with 1000 permutations. Importantly, module genes were pre-ranked by WGCNA intramodular connectivity to place greater weight on high connectivity hub genes during enrichment analysis. A less stringent threshold of an FDR-corrected q-value < 0.25 was chosen for significantly enriched gene sets for purposes of data exploration, per published recommendations.^4^

As secondary analysis, we identified specific cellular pathways underlying the functional terms identified in GSEA using the Leading Edge Analysis (LEA) feature, which identifies the subset of high-ranked genes within an input gene set that contributes most to each enrichment signal. As follow-up on GSEA results, LEA subsets were identified for top enrichment signals of interest, and the union of these 20 LEA subsets was used for correlation analysis (Turquoise module) or Enrichr enrichment analysis (Magenta module). In Enrichr, an unweighted list of LEA genes was queried for analysis against four databases: 1. ENCODE and ChEA Consensus TFs from ChIP-X (2016),^10–12^ 2. NIH Roadmap Epigenomics Histone Modification ChIP-seq data set (2015),^13,14^ 3. KEGG’s cell signaling pathways (2016),^15^ and GO terms for Biological Processes, Molecular Function, and Cellular Component (2018).^7,8^

***Gene-level analysis: qRT-PCR validation of select genes***

Total RNA was extracted as described above, and ~1 ug total RNA was used for cDNA synthesis using the Applied Biosystems High-Capacity cDNA Reverse Transcription Kit (cat. no. 4368814). TaqMan qRT-PCR assays were performed using the following probes: *ACTB*: Hs01060665_g1, *NUCB1*: Hs00939167_m1, *DHCR7*: Hs01023087_m1, *GOLGB1*: Hs00189566_m1, *PPP2R5D*: Hs00605059_m1 (cat. no. 4448490, 4331182). qRT-PCR reactions were conducted in triplicate on the 7900HT Fast Real-Time PCR System (Applied Biosystems) with polymerase activation at 95 °C for 10 min, and amplification at 95 °C for 15 s and 60 °C for 1 min for 40 cycles. Relative expression levels were calculated using the ΔΔCT method using beta-actin (*ACTB*) as the reference gene.^16^

***Tg challenge assays: Quantification of s/u XBP1***

Spliced-to-unspliced *XBP1* ratios (*s/u XBP1*), a marker of ER stress response activation, were quantified via qRT-PCR in triplicate using primers specific for spliced and unspliced XBP1 transcripts as specified by Oslowski et al.^17^ Reaction mix consisted to 5 uL SYBR Select Master Mix (2x), 1 uL of each primer (2 uM), 1 uL cDNA template, and 2 uL nuclease-free water for a total reaction volume of 10 uL. The following cycling settings were used: polymerase activation at 95 °C for 10 min, and amplification at 95 °C for 10 s and 58 °C for 30 s for 40 cycles. Relative levels of *s/u XBP1* were calculated using the ΔΔCT method.^16^

**References**

1. Kim, D. *et al.* TopHat2: accurate alignment of transcriptomes in the presence of insertions, deletions and gene fusions. *Genome Biol.* **14**, R36 (2013).

2. Liao, Y., Smyth, G. K. & Shi, W. The Subread aligner: fast, accurate and scalable read mapping by seed-and-vote. *Nucleic Acids Res.* **41**, e108 (2013).

3. Langfelder, P. & Horvath, S. WGCNA: an R package for weighted correlation network analysis. *BMC Bioinformatics* **9**, 559 (2008).

4. Subramanian, A. *et al.* Gene set enrichment analysis: a knowledge-based approach for interpreting genome-wide expression profiles. *Proc. Natl. Acad. Sci. U.S.A.* **102**, 15545–15550 (2005).

5. Chen, E. Y. *et al.* Enrichr: interactive and collaborative HTML5 gene list enrichment analysis tool. *BMC Bioinformatics* **14**, 128 (2013).

6. Kuleshov, M. V. *et al.* Enrichr: a comprehensive gene set enrichment analysis web server 2016 update. *Nucleic Acids Res.* **44**, W90-97 (2016).

7. Gene Ontology Consortium. Creating the gene ontology resource: design and implementation. *Genome Res.* **11**, 1425–1433 (2001).

8. Gene Ontology Consortium. Gene Ontology Consortium: going forward. *Nucleic Acids Res.* **43**, D1049-1056 (2015).

9. Liberzon, A. *et al.* Molecular signatures database (MSigDB) 3.0. *Bioinformatics* **27**, 1739–1740 (2011).

10. ENCODE Project Consortium. The ENCODE (ENCyclopedia Of DNA Elements) Project. *Science* **306**, 636–640 (2004).

11. Lachmann, A. *et al.* ChEA: transcription factor regulation inferred from integrating genome-wide ChIP-X experiments. *Bioinformatics* **26**, 2438–2444 (2010).

12. Rouillard, A. D. *et al.* The harmonizome: a collection of processed datasets gathered to serve and mine knowledge about genes and proteins. *Database (Oxford)* **2016**, (2016).

13. Bernstein, B. E. *et al.* The NIH Roadmap Epigenomics Mapping Consortium. *Nat. Biotechnol.* **28**, 1045–1048 (2010).

14. Roadmap Epigenomics Consortium *et al.* Integrative analysis of 111 reference human epigenomes. *Nature* **518**, 317–330 (2015).

15. Ogata, H. *et al.* KEGG: Kyoto Encyclopedia of Genes and Genomes. *Nucleic Acids Res.* **27**, 29–34 (1999).

16. Livak, K. J. & Schmittgen, T. D. Analysis of Relative Gene Expression Data Using Real-Time Quantitative PCR and the 2−ΔΔCT Method. *Methods* **25**, 402–408 (2001).

17. Oslowski, C. M. & Urano, F. Measuring ER stress and the unfolded protein response using mammalian tissue culture system. *Meth. Enzymol.* **490**, 71–92 (2011).
