# Supplementary figures and images for: Altered estradiol-dependent cellular Ca2+ homeostasis and endoplasmic reticulum stress response in Premenstrual Dysphoric Disorder
Source: Mol Psychiatry. 2021 May 25;26(11):6963–74. doi: 10.1038/s41380-021-01144-8 (PMC8613306; doi:10.1038/s41380-021-01144-8)

**Supplemental Figure 1: Experimental and analytic workflow**

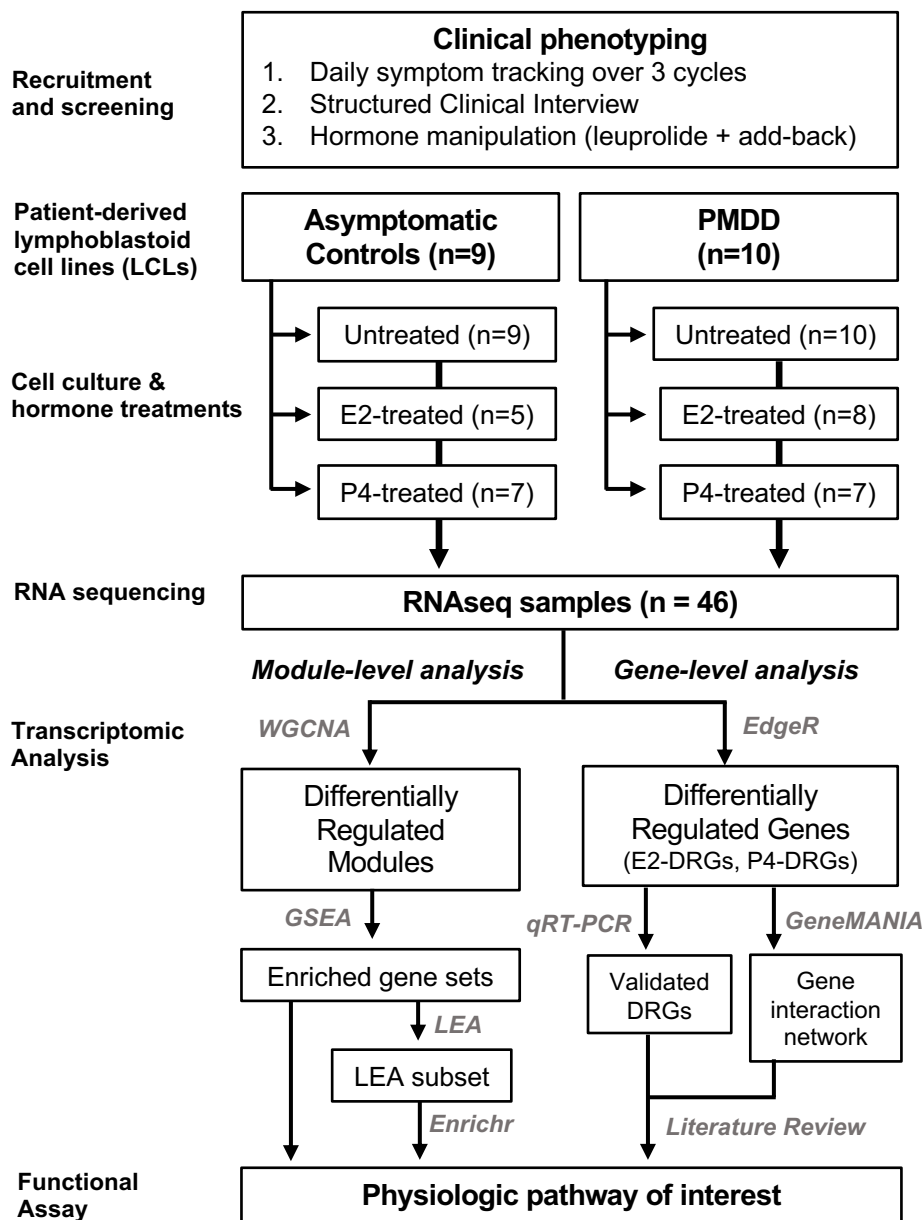

Supplement: Supplementary file 4 — Figure S1 [file 41380_2021_1144_MOESM4_ESM.pdf]
